# Aerobic Training for Healthy Men and Women: Determining Intensities by Different Equations

**DOI:** 10.3390/ijerph191912862

**Published:** 2022-10-08

**Authors:** Fernando Policarpo Barbosa, Andre M. Oliveira, Claudio Hernández-Mosqueira, Gustavo Pavez-Adasme, Pablo Luna-Villouta, Jairo Azocar-Gallardo

**Affiliations:** 1 Laboratory of Bioscience of Human Movement, Federal University of Rio de Janeiro, Rio de Janeiro 21941-901, Brazil; 2 Undersecretariat for Welfare and Quality of Life of the Government of the Federal District, Annex of the Palacio do Buriti, Brasilia 70075-900, Brazil; 3 Departamento de Educación Física, Deportes y Recreación, Universidad de La Frontera, Temuco 4780000, Chile; 4 Grupo de investigación AFSYE, Universidad Adventista de Chile, Chillan 3820572, Chile; 5 Facultad de Educación, Pedagogía en Educación Física, Universidad San Sebastián, Concepción 4030000, Chile; 6 Departamento de Ciencias de la Actividad Física, Universidad de Los Lagos, Puerto Montt 5480000, Chile

**Keywords:** prescription, aerobic exercise, healthy, intensities

## Abstract

The aim of this study is to develop equations for aerobic exercise prescription for the intensities of 50, 60, 70 and 80% in healthy subjects of both sexes. Method. This is a cross-sectional study with convenience sampling drawn from a database of 228 healthy subjects who were randomized into the regression group (GR: 197 subjects (male = 143 and female = 54)) and cross-validation group (CVG: 31 individuals [men = 20 and women = 11]). Kohavi’s assumptions were followed in relation to cross-validation and bootstrap for precision estimation and model selection. The GR was used to build the estimation equations from the multiple linear regression. The CVG was determined to analyze the validity in the estimation equations. The equations to determine the intensities were constructed by means of multiple regression, the independent variables were determined by the stepwise method, observing the significance level of *p* < 0.05. Results. The reliability level of Cronbach’s alpha of the multiple linear regression equations was moderate for the intensity of 50% (0.51); for the intensities of 60, 70 and 80%, it corresponded to 0.50, 0.53 and 0.57, respectively. Conclusion. The results show that it is possible to apply the equations in the determination of aerobic exercise intensities for healthy individuals. However, the need for further studies in other populations to prove the reliability of the proposed equations is evident.

## 1. Introduction

For decades, we sought to understand the physiological mechanisms during different levels of physical effort. For that, the use of mathematical models that make it possible to estimate the metabolic adjustments resulting from the workloads imposed during physical exercise have become a recurrent non-invasive procedure widely used in studies [1,2,3]. Understanding the interaction between the different cardiovascular, respiratory and anthropometric systems allows the development of models to estimate the intensities for physical exercise [4,5,6].

In the specific case of aerobic exercise, the association observed between training intensities and oxygen consumption [6,7] made it possible to develop estimation equations such as those of Karvonen et al. [8] and Swain [9]. However, in recent decades, questions have arisen about the linearity between oxygen consumption (VO_2_) and heart rate (Hr) in the determination of aerobic exercise intensities [10,11]. In the studies by Rolnick and Schoenfeld [12] and Peters, et al. [13], the methods of Karvonen et al. [8] and Swain [9] tended to underestimate or overestimate, respectively, the intensities of the aerobic exercises. Therefore, Rolnick and Schoenfeld [12] and Peters, et al. [13] suggested developing estimation equations for each aerobic training intensity to obtain greater accuracy in the prescription of aerobic exercise intensities, as proposed by the American College of Sports Medicine [14].

Peters, et al. [13] sought to base his suggestion on the mechanisms (chemoreceptors, baroreceptors and mechanoreceptors) related to HR control during exercise, as described by Nóbrega, et al. [5]. However, there are other factors that have a close relationship with VO2 and the intensity of aerobic exercise. Herdy and Uhlendorf [15] and Loe, et al. [16] observed a significant reduction in cardiorespiratory fitness with age, pointing out that both age and physical conditioning are determinant variables that affect the response to physical exercise [6].

Given the above, it would be possible to develop specific equations for each intensity using age as the intensity estimation variable [12,13], as well as other variables related to cardiopulmonary fitness. How would it be possible to not use the estimated maximum heart rate (HRmax) as a parameter in the intensities calculations? The HRmax tends to underestimate or overestimate the intensity of the aerobic training when correlated with the percentages of maximum oxygen consumption [7,10]. In view of the hypotheses presented, the present study aims to develop and validate equations for estimating the intensities of 50, 60, 70 and 80% of peak oxygen consumption for prescribing aerobic training for healthy individuals of both sexes.

## 2. Materials and Methods

### 2.1. Study Design

**Figure 1:**
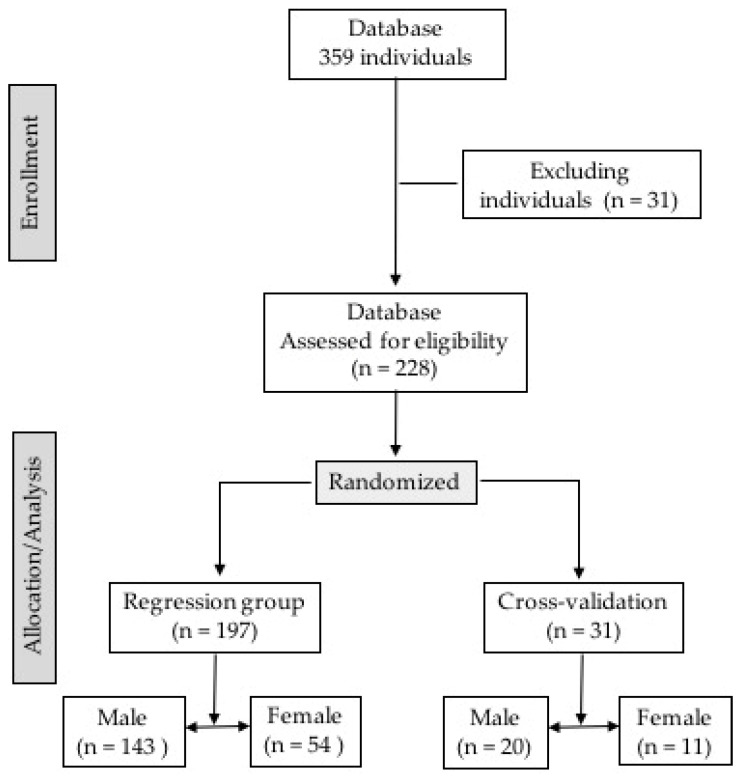
Description of the selection process and randomization of regression and cross-validation groups.

This is a cross-sectional study with convenience sampling extracted from a database of 259 subjects, of which 31 subjects were excluded from the study because they did not complete the cardiopulmonary exercise test. The 228 remaining subjects were randomized into two groups using the randomization function of the SPSS statistical software package version 19 (IBM, Chicago, IL, USA) as follows: (a) regression group (GR), composed of 197 subjects (men = 143 and women = 54) and (b) individuals in the cross-validation group (CVG), with 31 individuals (men = 20 and women = 11) (Figure 1). This study was approved by the Ethics Committee of the Catholic University of Brasília (UCB), Brasília, DF, Brazil, protocol 045/2005.

The RG was used to construct the estimation equations from multiple linear regression. The CVG was determinate for analyzing the validity in the estimation equations. Therefore, assumptions by Kohavi [1] were followed regarding cross-validation and bootstrap for accuracy estimation and model selection. Thus, the CVG sample size was defined following the assumptions described by Mourão [17], which demonstrated that 30 patients could favor a normal distribution curve. Initially, all the subjects were informed of the study aims, as well as the possible risks and benefits of the experiment. Next, subjects were provided with informed consent and underwent clinical examination and rest electrocardiogram (ECG); if a problem was identified, the individual was excluded from the study. All subjects were submitted to the following anthropo-metric measurements: (a) body mass, obtained on a digital scale (Filizola^®^, São Paulo, Brazil), accurate up to 100 g, and (b) height using a stadiometer (Seca-Country Technology^®^ 67034, Benson Avenue, CA, USA), with the scale set to centimeters. The Jackson and Pollock [18] protocol was used to estimate body density in men, and the three-point skin-fold method, as described by Jackson et al. [19], was used in women. Body fat was estimated using the Sire protocol [20].

### 2.2. Incremental Degree CPET Protocol

An incremental degree CPET protocol was conducted using the Super-ATL^®^ treadmill (Inbramed, Porto Alegre-Brazil) until the maximal effort (respiratory coefficient ≥ 1.1) as recommended by ATS/ERS CPET [21]. Every incremental degree had a duration of 1 min. The initial velocity was 4 km/h with 0% incline. The velocity and inclination were increased every minute by 1 km/h and 0.5%, respectively. The maximal velocity was 16 km/h, and the maximal inclination was 6%. This standard protocol for individuals of both sexes classified as sedentary and/or moderately active used in the laboratory, which was published later [22]. This protocol demonstrated that these adjustments could better fit a maximal test of 8–12 min in healthy subjects as has been suggested by the ATS statement [21]. Ventilatory-expired gas analysis was obtained using a VO2000^®^ gas analyzer (Aerosport Medgraphics, Saint Paul, MN, USA), with gas samples collected every 10 s. The gas analyzer was calibrated before the tests with a known gas: 17% oxygen, 5% carbon dioxide gas and nitrogen balance. Heart rate was recorded in CM5 derivation, using a digital electrocardiogram (Micromed, Brasilia, Brazil). The treadmill, electrocardiogram and gas analyzer were linked to the Ergo-PC Elite^®^ software (Micromed, Brasilia, Brazil).

Procedure to determine the percentages of Heart rate relative to the consumption of oxygen (HR/VO_2_).

After CPET, the intensities of 50, 60, 70 and 80% of peak oxygen consumption (VO_2peak_) were calculated. As described in the studies by Rolnick and Schoenfeld [12] and Peters, et al. [13], the mean of the VO_2peak_ for the respective intensities was calculated for the corresponding minute, as well as the heart rate of the same interval (HR/VO_2_).

### 2.3. Statistical Analysis

Statistical treatments were performed using the SPSS package version 19 (IBM, Chicago, IL, USA). The presentation of the results will be by the mean and standard deviation (±SD). For the development and cross-validation of the estimation equations, statistical analyzes were applied: (a) Normality test: Kolmogorov–Smirnov and QQ graphs [23]; (b) stepwise multiple linear regression “forward” [24]; (c) comparison between the percentages of HR/VO_2_ measured and estimated by the equations developed by the paired *t* test; (d) Pearson correlation and reliability analysis using Cronbach’s alpha; and (e) residual Bland and Altman scores. The Standard Error of Estimate (SEE) adopted as a criterion of acceptance and validity of the estimation equation was <10 beats per minute (bpm). The significance level adopted was *p* < 0.05.

## 3. Results

All volunteers were considered healthy and released by the laboratory doctor to perform the cardiopulmonary exercise test. The tests were carried out in the morning and afternoon, the average speed was 13.4 ± 2.0 km/h with an inclination of 3.0 ± 1.0% with a duration of 9.3 ± 4.5 min and the temperature varied between 20 °C in the morning and 24 °C in the afternoon, with relative humidity between 50% and 60%. The anthropometric characteristics of the sample are shown in Table 1.

The resting heart rate (HRrest) was one of the independent variables for the construction of the straight line. In the case of women in the RG, the mean HRrest was 65.7 ± 8.0 bpm, the mean VO_2_peak = 42.6 ± 6.7 mL/kg·min^−1^ and mean HRmax = 187.9 ± 7.8 bpm. In the male RG, the mean resting HR = 64.9 ± 10.8 bpm, mean VO_2_peak = 55.6 ± 9.0 mL/kg.min^−1^ and mean HRmax = 190.3 ± 9.6 bpm. The ‘‘forward add’’ method indicated age as the other independent variable in the composition of the straight line, and the other variables, body mass, height, body fat percentage and HRmax, were discriminated.

The construction of the regression line for the respective intensities was significant. However, the values of R^2^ demonstrate that the independent variables age and HRrest explain approximately 50% of the construction of the line (Table 2), which is convergent with the moderate level of reliability of the equations determined by Cronbach’s alpha. For the 50% intensity, Cronbach’s alpha = 0.51, and for the 60, 70 and 80% intensities, it was 0.50, 0.53 and 0.57, respectively. However, the standard error of the estimate was less than 10 bpm (SEE mean = 6.3 bmp).

**Figure 2:**
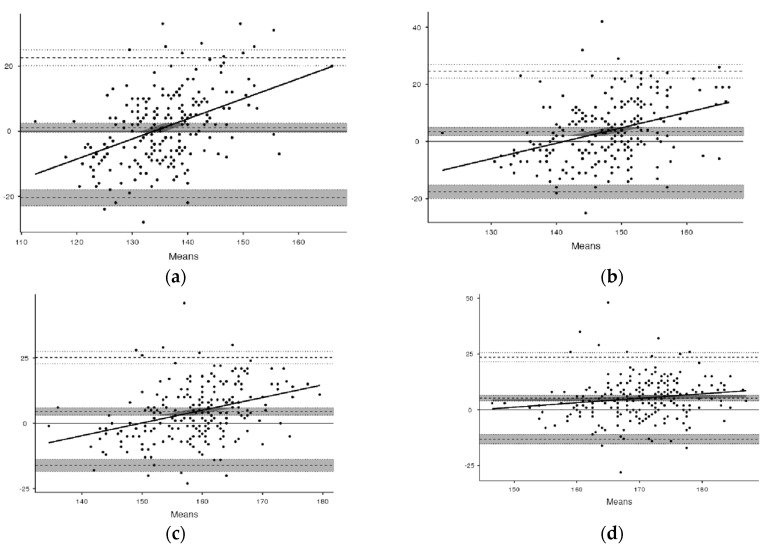
Analysis of Bland and Altman residual scores between HR/VO_2_ and estimation equations for intensity of 50, 60, 70 and 80% VO_2peak_ for 197 healthy individuals of both sexes. (**a**) 50% of HC/VO_2peak_. (**b**) 60% of HC/VO2peak. (**c**) 70% of HC/VO_2peak_. (**d**) 80 of HC/VO_2peak_.

The comparison of the values estimated by the equations in relation to the reference values HR/VO_2_ (Table 3) showed statistically significant differences *p* > 0.05 in the intensities of 60, 70 and 80%. However, it is worth mentioning that the standard deviation of HR/VO_2_ (reference value) was on average 12 bpm while the equations had an average of 4 bpm. The mean of 12 bpm indicates high heart rate variability for the same percentage of oxygen consumption, as can be observed by residual stresses (Figure 2). The anthropometric characteristics of the cross-validation group can be observed in Table 4.

For the cross-validation process, that is, the application of the equations to estimate the intensities of 50, 60, 70 and 80% of VO_2peak_, in a different sample, the cross-validation group (CVG), showed a significant statistical difference (*p* > 0.05) for the intensities of 50% and 60%. However, for the intensities of 70% and 80%, there was no statistical difference in Table 5.

## 4. Discussion

The present study sought, by means of multiple linear regression, to develop equations to estimate the intensities of 50, 60, 70 and 80% of VO_2peak_, as an alternative for the prescription of aerobic exercise. The results obtained demonstrated that the equations can be a plausible alternative for the prescription of aerobic exercises in healthy young people.

The behavior of the data as a function of the regression lines indicates better adjustments for each age group and intensity, and this may be related to the adjustment of the constant for each intensity. What does not occur in the HRreserve equation is that the difference between HRmax and HRrest will be the same in the calculation of the different intensities, which can explain the smaller dispersion of the Standard Error of Estimation in each intensity.

The statistical differences observed mainly in the 50% and 60% intensities are due to the deviation greater than 12 bpm for HR/VO_2peak_, which explains the tendency of the equations to underestimate the reference values. According to Nobrega et al. [5], the observed variability may be due to the Frank–Starling mechanism and the modulations in the autonomous system [23]. Kisaka et al. [3] cite the intrinsic physiological mechanisms in the control of heart rate, which presents greater parasympathetic dependence for this level of effort [5].

The interaction between the different physiological mechanisms is undoubtedly the determining factor in the design of the estimation equations. Since resting heart rate can be affected by different external factors [12,13], Peters et al. [13] observed that the measurement of resting heart rate in different positions results in a significant difference in the determination of intensities. Therefore, resting heart rate is a physiological component that allows characterizing both biological individuality and cardiopulmonary fitness level [6,24,25]. This peculiarity may explain the conclusion Engels, Zhu, Moffatt [26] that contest the simple application of the percentages of the HRmax estimated by the equation 220 –age to determine the intensities of the aerobic training.

In the specific case of the VO_2_R method, the estimated consumption of oxygen at rest (1 MET) is used. Studies that sought to verify the relationship between the percentages of VO_2_R and VO_2max_ showed a tendency to overestimate the intensities corresponding to VO_2max_ [12,27]. The explanation may be related to the use of MET; this consumption represents conditions of basal metabolism and not at rest [28]. Two studies that measured oxygen consumption in the orthostatic position for five minutes had mean values greater than 3.5 mL/kg·min^−1^ [28,29].

The other method for determining intensities is the resting heart rate (HRR) equation, which follows the same principle of incorporating an individual component with resting HR. However, this method is combined with other HRmax estimation equations. However, recent studies have shown that HR is an excellent physiological parameter for controlling external loads [5,30,31].

This converges with the results obtained in the present study, reinforcing the importance of equations that seek better adjustments in the estimation of aerobic exercise intensities. Therefore, we used the data described in the study of Eriksson et al. [30], which had a sample of 34 individuals of both sexes with a mean age of 44 years and resting HR = 59 bpm. With this information, the intensities of 50 and 60% of VO_2_ were calculated by the equations developed in the present study, obtaining for 50% = 132 vs. 131 bpm and for 60% = 139 vs. 143 bpm. The equations proposed in this study, even when applied to the population and in different climatic conditions, present similar results. The equations presented try to fill the gap described in the studies by Policarpo, et al. [27] and Rolnick and Schoenfeld [12], who observed the need for specific equations for each intensity instead of general equations [7,9,28].

### Limitations and Strengths

Within the limitations of this study, it is possible to highlight the application of the equations in middle-aged and elderly individuals, which implies the need for further studies to prove its accuracy in all age groups, even though the correction factor for age is observed in the equations developed. At the same time, the equations presented cannot be applied to patients with heart disease or other diseases without proving their applicability to this population.

## 5. Conclusions

The equations proposed in the present study to estimate the intensities of 50, 60, 70 and 80% of the VO_2peak_ of active and sedentary young individuals aged between 17 and 46 years old showed a moderate level of reliability = 0.5 with the Standard Error of Estimation less than 10 bpm. These results allow us to accept the hypothesis of the study, demonstrating that it is possible to develop specific equations for each intensity mentioned above. However, there is a need for further studies in other elderly populations.

## Figures and Tables

**Table 1:** Values Mean and standard deviation (SD) of the regression group (RG) for the anthropometric and age of individuals of both sexes.

	Men (*n* = 143)	Women (*n* = 54)	General (*n* = 197)
Age	25.4 ± 6.4	22.2 ± 4.7	24.6 ± 6.2
Height (cm)	175.0 ± 6.4	162.4 ± 6.5	171.0 ± 8.6
Body mass (kg)	73.2 ± 10.0	57.1 ± 8.4	68.7 ± 11.8
BMI (kg/m^2^)	23.9 ± 2.9	21.6 ± 2.7	23.3 ± 3.0
% body fat	13.2 ± 6.4	20.1 ± 6.3	15.2 ± 7.1

**Table 2:** Equations developed to estimate the intensities of 50, 60, 70 and 80% for the regression group (GR) having the HR/VO2 measured in a treadmill exercise test as the dependent variable.

Equations	R	R^2^	SEE	*p* Value
HR_t 50%_ = 93.6 + (HR_rest_ × 0.6) − (age × 0.2)	0.69	0.48	5.4	*p* = 0.01
HR_t 60%_ = 111.9 + (HR_rest_ × 0.5) − (age × 0.2)	0.58	0.34	6.0	*p* = 0.01
HR_t 70%_ = 130.7 + (HR_rest_ × 0.4) − (age × 0.2)	0.51	0.26	6.6	*p* = 0.01
HR_t 80%_ = 149.6 + (HR_rest r_ × 0.3) − (age × 0.3)	0.42	0.18	7.2	*p* = 0.01

HR_rest_ = resting heart rate in the orthostatic position. SEE = standard error of estimate.

**Table 3:** Mean values and standard deviation of HR/VO_2_ measured and HR estimated by the proposed equations for intensities of 50, 60, 70 and 80% for the regression group (GR).

	HR/VO_2_	Equations
HR_t50%_	135.7 ± 12.4	135.3 ± 4.7
HR_t60%_	148.8 ± 12.6	146.4 ± 4.1 **
HR_t70%_	161.0 ± 12.0	157.5 ± 3.7 **
HR_t80%_	172.6 ± 11.0	169.0 ± 3.7 **

HR_t50%_ = values for intensity of 50% of VO_2peak_; HR_t60%_ = values for intensity of 60% of VO_2peak_; HR_t70%_ = values for intensity of 70% of VO_2peak_; HR_t80%_ = values for intensity of 50% of VO_2peak_; HR/VO_2_ = heart rate values corresponding to oxygen consumption; Equations = values for the intensities determined by the equations with reference to the HRmax measured in test; ** *p* < 0.01.

**Table 4:** Anthropometric characteristics of individuals of both sexes (*n* = 31) in the cross-validation group.

	Men (*n* = 20)	Women Woman (*n* = 11)
Age	28.0 ± 6.7	29.0 ± 8.6
Height (cm)	175.1 ± 5.3	162.0 ± 8.3
Body mass (kg)	77.1 ± 13.0	60.0 ± 9.8
BMI (kg/m^2^)	25.0 ± 3.3	23.2 ± 3.3
% body fat	14.1 ± 6.7	25.1 ± 5.5

**Table 5:** Estimated values for the intensities of 50, 60, 70 and 80% of aerobic training compared to reference values (HR/VO_2_) for individuals of both sexes in the cross-validation group (CVG).

	HR/VO_2_ (bpm)	HR _equation_ (bpm)	SEE (bpm)
HR_t50%_	129.6 ± 13.1	134.6 ± 7.6 *	2.1
HR_t60%_	140.5 ± 12.8	145.4 ± 6.5 *	2.1
HR_t70%_	154.1 ± 11.2	156.1 ± 5.6	2.0
HR_t80%_	165.1 ± 11.5	166.7 ± 5.1	2.0

HR_t50%_ = values for intensity of 50% of VO_2peak_; HR_t60%_ = values for intensity of 60% of VO_2peak_; HR_t70%_ = values for intensity of 70% of VO_2peak_; HR_t80%_ = values for intensity of 80% of VO_2peak_; HR/VO_2_ = heart rate values corresponding to the percentage of oxygen consumption; * *p* < 0.05.

## Data Availability

The database is not available for direct access, but can be requested from researchers.
